# Prediction of movement intention using connectivity within motor-related network: An electrocorticography study

**DOI:** 10.1371/journal.pone.0191480

**Published:** 2018-01-24

**Authors:** Byeong Keun Kang, June Sic Kim, Seokyun Ryun, Chun Kee Chung

**Affiliations:** 1 Human Brain Function Laboratory, Department of Neurosurgery, Seoul National University Hospital, Seoul, South Korea; 2 Interdisciplinary Program in Neuroscience, Seoul National University College of Natural Science, Seoul, South Korea; 3 Department of Brain & Cognitive Sciences, Seoul National University College of Natural Sciences, Seoul, South Korea; 4 Department of Neurosurgery, Seoul National University College of Medicine, Seoul, South Korea; Universitair Medisch Centrum Groningen, NETHERLANDS

## Abstract

Most brain-machine interface (BMI) studies have focused only on the active state of which a BMI user performs specific movement tasks. Therefore, models developed for predicting movements were optimized only for the active state. The models may not be suitable in the idle state during resting. This potential maladaptation could lead to a sudden accident or unintended movement resulting from prediction error. Prediction of movement intention is important to develop a more efficient and reasonable BMI system which could be selectively operated depending on the user’s intention. Physical movement is performed through the serial change of brain states: idle, planning, execution, and recovery. The motor networks in the primary motor cortex and the dorsolateral prefrontal cortex are involved in these movement states. Neuronal communication differs between the states. Therefore, connectivity may change depending on the states. In this study, we investigated the temporal dynamics of connectivity in dorsolateral prefrontal cortex and primary motor cortex to predict movement intention. Movement intention was successfully predicted by connectivity dynamics which may reflect changes in movement states. Furthermore, dorsolateral prefrontal cortex is crucial in predicting movement intention to which primary motor cortex contributes. These results suggest that brain connectivity is an excellent approach in predicting movement intention.

## Introduction

Volitional movement prediction has become an area of focus of brain-machine interface (BMI) research. Three-dimensional movements have been successfully controlled by BCI models using the primary motor area activities in humans and primates [1–3]. The prediction models implicated the motor parameters of hand position, velocity, and force as crucial during movement. The conventional experimental paradigm for motor control consists of the active period and the idle period. The active period is the time to perform the movement task. The idle period is the rest time.

Most movement prediction studies have focused on the active period. Since the parameters are optimized for the moving state, they may be maladapted in the idle state [4]. In the real world, robot arm prosthesis driven by the motor cortex activity must always be precise. If not, the prediction could error unexpectedly during the idle period. One possible way to prevent unexpected error is to know when movement is intentional.

A full-time-prediction model is practically very difficult and inefficient because conventional movement prediction models have been optimized only for the active period and feature kinematic parameters obtained from active period data. Alternatively, a BMI system could selectively be operated when the user intends, which is a more efficient and reasonable system. To move towards the latter scenario, electrophysiology has been extensively investigated as a means of predicting the movement onset time [5–9] or intention [4, 10, 11]. Previous studies only used local signal characteristics derived at individual electrodes, a variety of signal features were used including the local motor potential as well as spectral power in various frequency bands to optimize a decoder for different movement states [12–14]. However, in real life, people progress through several states to achieve a behavior goal. Movement intention is generated by the change of these states. The sensorimotor system comprises motor planning, motor command generation, state transition, and sensory feedback generation [15]. All these states involve complicated processes that harness functionally different brain areas. Even though power spectral analysis is a good tool to analyze signal characteristics, it is limited for the analysis of the relationship of brain areas. Therefore, there may be some improvement in model performance using information transfer among brain areas compared to the conventional method.

Based upon scientific findings and the information gleaned from previous studies, the best choice would be the introduction of brain connectivity dynamics. An emerging means of improving BMI is the brain network [16, 17]. Brain network studies have included motor, language, and memory in neuroimaging and electrophysiology [18–20]. With regard to the motor network, recent evidence suggests that movements arise from the distributed networks that center on primary motor cortex (1M, 1M is used to refer primary motor cortex instead of M1 to avoid confusion with mutual information (MI)) [21]. The dorsolateral prefrontal cortex (DLPFC) plays a crucial role to exert control over behavior, and it has a central integrative function used for motor control and behavior [22–25]. The motor-related areas then receive signals from the DLPFC and posterior parietal cortex, which help mediate movement [26].

Based on these findings, we assume that the DLPFC and 1M is closely connected by information transmission with different connectivity dynamics over the movement time. Connectivity dynamics can predict the change in movement states whether a subject intends to move or not. In this study, our aim is to predict the movement intention using network dynamics represented by an intra- and inter-regional connectivity in DLPFC and 1M areas. To do this, we used subdural electrocorticography (ECoG) covering DLPFC and 1M.

## Materials and methods

### Ethics statement

Prior to the study, all subjects submitted written informed consent for participating in the study. This was approved by the Institutional Review Board of the Seoul National University Hospital (IRB number: H-0912-067-304).

### Subjects

Three epilepsy patients participated in this study (Table 1). All subjects underwent implantation of subdural electrodes over the 1M and DLPFC. 1M electrodes were identified by electrical stimulation during surgery. DLPFC electrodes were determined based on the brain anatomy. All subjects were scanned using 3-T magnetic resonance imaging (MRI) (Verio; SIEMENS) and 3-D computed tomography (CT) (Sensation 16; SIEMENS) before and after subdural electrode implantations, respectively.

**Table 1:** Clinical profiles.

Subject	Sex	Age	Side of hand movement	Location of intracranial electrodes	
				Location	Number
1	M	28	Right	Left F, P, T	64
2	F	31	Right	Left F, P, T	64
3	F	36	Left	Right F, P, T	72

Abbreviations: F, frontal; P, parietal; T, temporal; O, occipital

### Experimental protocols and data acquisition

**Fig 1:**
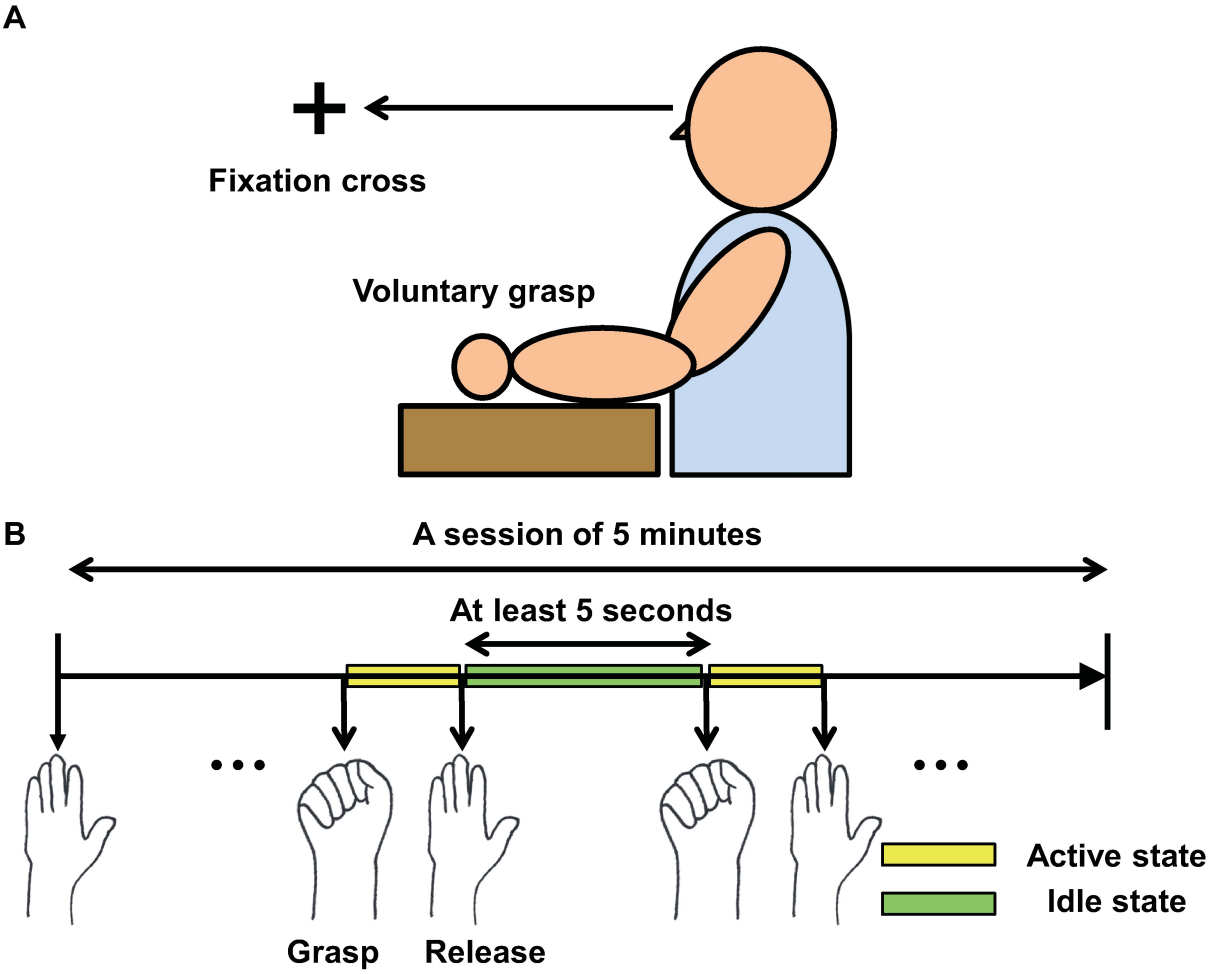
Experiment paradigm. (A) The subjects were seated comfortably on their bed with their arms supported on a table. During a session, subjects were instructed to fixate their gaze at a cross on the wall. (B) The subjects performed voluntary hand grasp in a session of 5 minutes. A grasp-to-grasp interval should be at least 5 seconds. Yellow box represents the active state during the time of performing grasp and green one represents the idle state during the time of resting.

All subjects were asked to repeatedly grasp and release the hand contralateral to the implanted hemisphere in accordance with approved guidelines (Fig 1). The subjects were instructed that a grasp-to-grasp interval should be at least 5 seconds. Subjects were also asked not to count the seconds so that they could keep gaze into a fixation cross. Subjects performed the task in a session of 5 minutes. There were 25–39 trials in a session. The individual variability of a number of trials was accrued to the difference in individual task speed.

Each subject had subdural electrodes (Ad-tech Medical Instrument, Racine, WI, USA). The number of electrodes was between 68 and 72. The subdural electrodes were stainless steel discs of 4 mm in diameter. The inter-electrode distance was 10 mm. Implanted electrodes and individual brain model were reconstructed from the individual MRI and CT images by using CURRY version 7.0 software (Compumedics Neuroscan, Charlotte, NC, USA). ECoG activity was recorded using a 128-channel Neuroscan synamps2 (Compumedics USA, Ltd., EL Paso, TX, USA), digitized at 1000 Hz per channel. Referential electrodes were placed on cheekbones.

During the experiment, the surface electromyography (sEMG) on the opponens pollicis in the contralateral hand was simultaneously recorded. The sEMG then was used to discriminate two movement states of the active state (AS) and idle state (IS). The period of movement execution will be termed as the AS and the period of resting will be termed as the IS. Electrooculography (EOG) was also recorded to minimize an effect of eye movement.

### Signal preprocessing

**Fig 2:**
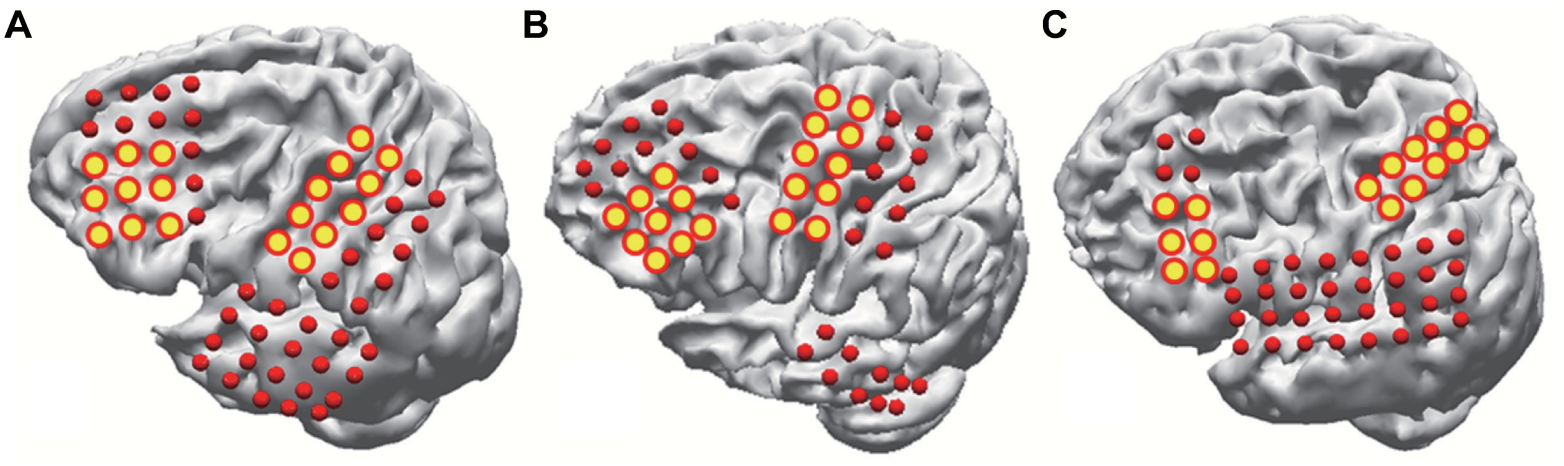
Electrode locations in the three subjects. All three subjects underwent implantation of subdural electrodes over the frontal, parietal, and temporal cortices. The analysis was conducted with yellow circled electrodes. (A) Subject 1. (B) Subject 2. (C) Subject 3.

ECoG data were analyzed using Matlab software (Mathworks, Natick, MA, USA). The ECoG was divided into two movement states. The movement states were determined based on the sEMG signals. We selected electrodes for further analysis which represented 1M and DLPFC (Fig 2). Common average references (CARs) were used to remove the global background activity on all the recorded channels [27]. After CARs, the ECoG data was filtered between 1–300 Hz, with a two-way least-squares finite impulse response filtering. A 60 Hz notch filter was also applied.

### Connectivity analysis

There were two kinds of connectivity to represent information transmission within distributed electrodes. Intra-connectivity referred to inward information transmission of a specific region. The 1M network indicated 1M intra-connectivity and DLPFC network indicated DLPFC intra-connectivity. Inter-regional connectivity referred to information transmission between two different regions. DLPFC-1M network indicated inter-regional connectivity of DLPFC and 1M. These networks demonstrated that how much information transmitted within a specific functional area or between two different areas.

Mutual information (MI) [28]was used to analyze intra-and inter-regional connectivity dynamics. MI is an excellent method to measure functional connectivity and a statistical measure of both linear and nonlinear dependencies between signals. For example, if the two electrode signals are independent, there is no shared information. Hence the value of MI is zero. However, when there is a dependency between two signals, the MI value is higher than zero. MI was calculated as follows:

$$MI=\sum P\left(X,Y\right)*\text{ln}\left(\frac{P\left(X,Y\right)}{P\left(X\right)*P\left(Y\right)}\right),$$ (1)

where P(*X*,*Y*) is the joint probability distribution function of variable *X* and *Y*, and P(*X*) and P(*Y*) are the marginal probability distribution functions of *X* and *Y*, respectively. Before calculating MI, we applied a band pass filter between 4–7 Hz for theta band, 8–13 Hz for the alpha band, 13–30 Hz for the beta band, and 30–50 Hz for gamma band. Then, we calculated MI with 8 bins in each frequency band. In order to continuously estimate the connectivity, we used 1 s windows shifting every 100 ms.

To investigate which frequency band shows a significant difference between AS and IS, we defined the mutual information ratio (MIR). The MIR represented the connectivity increase of AS compared to IS. The MIR in a specific brain network (MIR_network_) was calculated as follows:

$$MIR_{network}=\frac{1}{N}\overset{N}{\underset{p=1}{\sum }}\frac{\frac{1}{K}\sum_{a=1}^{K}MI_{AS}(p,a)-\frac{1}{L}\sum_{b=1}^{L}MI_{IS}(p,b)}{\frac{1}{L}\sum_{b=1}^{L}MI_{IS}(p,b)}\times 100\left(\%\right),$$ (2)

where *N* is the total number of connectivity pairs in a defined network such as an 1M network with intra-connectivity pairs, a DLPFC network with intra-connectivity pairs, and an 1M-DLPFC network with inter-regional connectivity pairs. *K* was the total number of time points in AS, and *L* was the total number of time points in IS.

### Classification

Linear discriminant analysis was used to classify movement intentions. The classifier was calibrated to distinguish between two classes (i.e. AS and IS) by the dynamics of intra- and inter-regional connectivity. According to sEMG signal, the training data were then labeled AS for the period of hand grasping and IS for the resting.

To estimate accuracy, we used a 10-fold cross-validation in which data were permuted and partitioned into 10 blocks of an equal size. In 10 blocks, nine blocks were used for training the classifier, and one remaining block was used for testing the accuracy. This procedure was repeated 10 times, and the accuracy was averaged over all folds.

## Results

Three epilepsy patients participated in this study (Table 1). They underwent implantation of subdural electrodes over the DLPFC and 1M (Fig 2). During the experiment, they were asked to repeatedly perform the task of a voluntary hand grasping with the contralateral hand of an implanted hemisphere (Fig 2A). We defined two different states according to the sEMG on the opponens pollicis. The AS indicated the period of movement and the IS indicated the period of resting (Fig 2B). The criteria for splitting AS and IS is defined as the time when the EMG activity exceeds a threshold equal to μ + 3σ, where μ and σ are the mean and standard deviation of EMG signals of a one-second window during resting. In connectivity analysis, we used the MI to investigate temporal connectivity dynamics and defined three networks: DLPFC, 1M, and DLPFC-1M networks.

### Connectivity dynamics between active state and idle state

**Fig 3:**
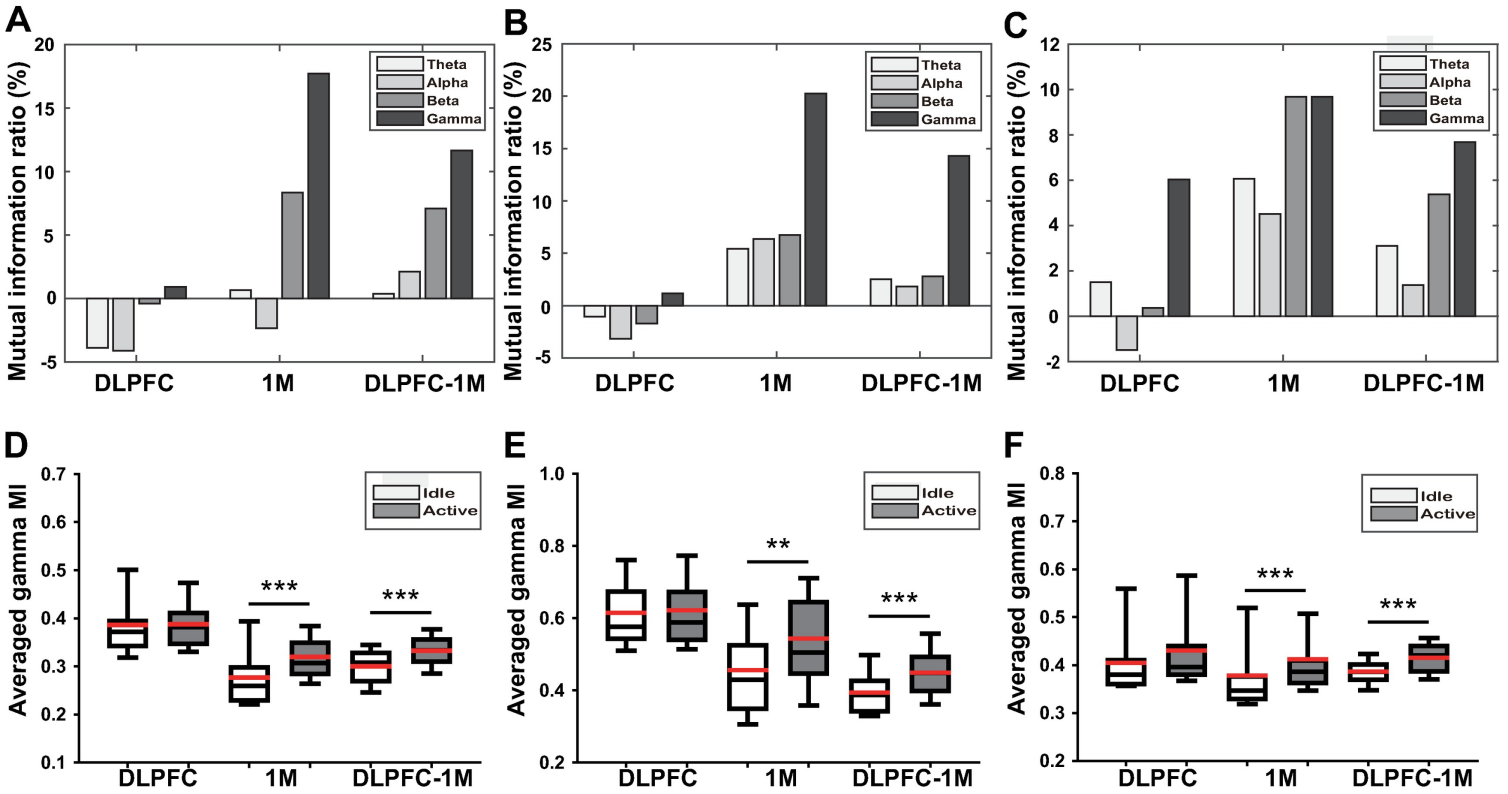
Measured connectivity changes. The beta and gamma results of MIR in the first row and the gamma band statistical test in the second row (1^st^ column: Subject 1, 2^nd^ column: Subject 2, 3^rd^ column: Subject 3). The first row indicates that the beta and gamma band connectivity increased in the active state compared to the idle state. The second row indicates the result of two-sample Kruskal-Wallis test for the gamma band, ** denotes p < 0.01 and *** denotes p < 0.001. It represented the averaged gamma MI value in the active and idle state, respectively (black line: median, red line: mean).

The MIR of beta and gamma bands were of prominent difference between AS and IS. The results of MIR on beta and gamma bands are shown in Fig 3 (first row). In all three subjects, the 1M network and the DLPFC-1M network increased in beta and gamma bands. The beta band connectivity increased 8.3% in the 1M network and 5.1% in the DLPFC-1M network on average. The gamma band connectivity increased 15.9% in the 1M network and 11.2% in the DLPFC-1M network on average. However, the DLPFC network slightly changed in both beta and gamma band compared to the 1M and DLPFC-1M networks. According to MIR results, the gamma band shows the most prominent MI increase in AS among four bands: theta (4–7 Hz), alpha (8–13 Hz), beta (13–30 Hz), and gamma bands (30–50 Hz). We performed a two-sample Kruskal-Wallis test for significance test of gamma band. The result demonstrated that MI of AS in 1M network and DLPFC-1M network was significantly higher than that of IS (Subject 1: 1M AS vs. 1M IS, N = 45, *p* < 0.0001, DLPFC-1M AS vs. DLPFC-1M IS, N = 90, *p* < 0.0001, Subject 2: 1M AS vs. 1M IS, N = 45, *p* = 0.0018, DLPFC-1M AS vs. DLPFC-1M IS, N = 90, *p* < 0.0001, Subject 3: 1M AS vs. 1M IS, N = 45, *p* = 0.009, DLPFC-1M AS vs. DLPFC-1M IS, N = 60, *p* < 0.0001). N denotes the number of all possible electrode pairs in the network). However, the DLPFC network didn’t show significant change between AS and IS. Based on these results, we decided to use the gamma frequency band as a target frequency band to predict movement intention.

### Temporal dynamics of intra- and inter-regional connectivity

**Fig 4:**
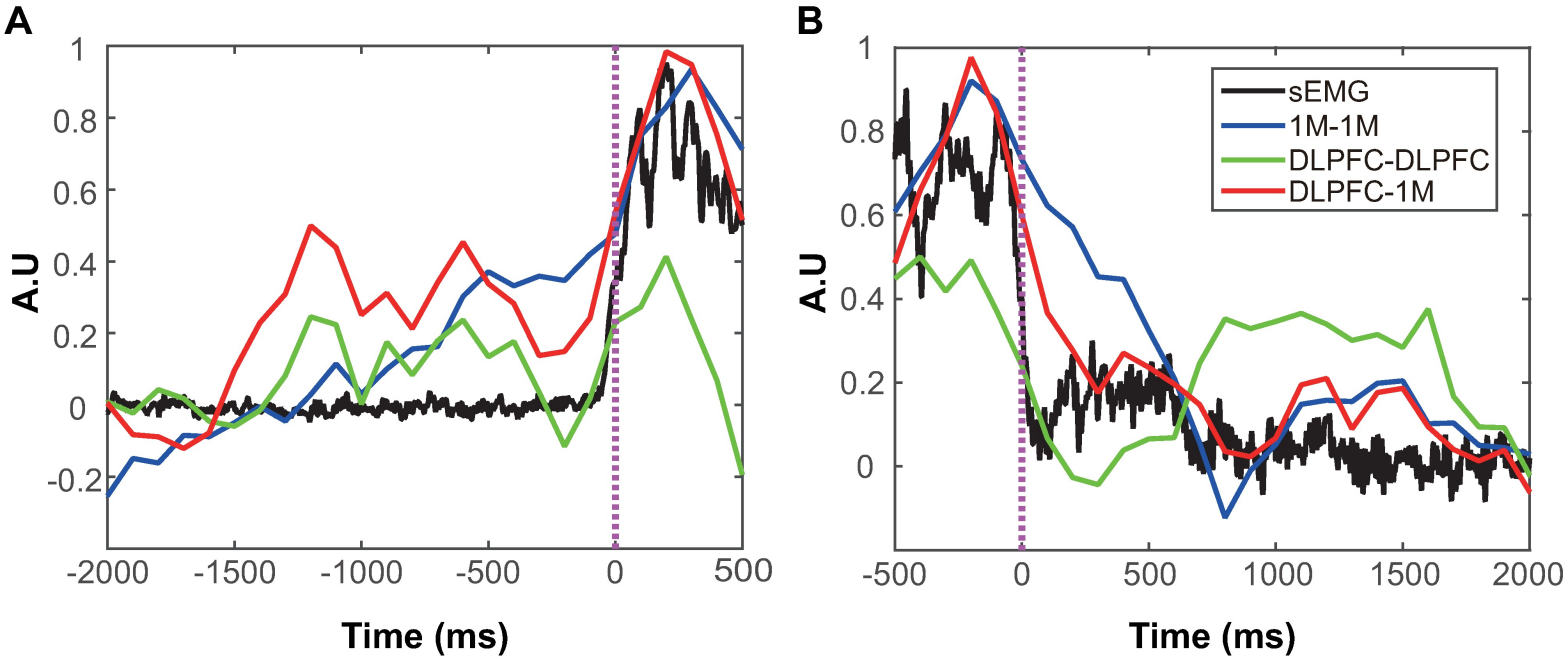
Temporal connectivity dynamics in DLPFC, 1M, and DLPFC-1M networks. Temporal connectivity dynamics are represented by the onset and offset of the movement, respectively. Pink vertical dotted line denotes movement onset (A) and offset (B).

The temporal connectivity evolutions in DLPFC, 1M, and DLPFC-1M networks show the information flow at each state such as idle, movement planning, execution, and recovery. To investigate the connectivity evolution, MI values of each pair were normalized between 0 and 1 by means of the minimal and maximal MI values across a session. We demonstrated the results of two separate periods, onset and offset, since the duration of grasp varied with every trial. To do this, we extracted epochs between −2000 ms and 500 ms of the movement onsets for grasp, and epochs between −500 ms and 2000 ms of the movement offsets for release. All epochs were then averaged across trials. The temporal connectivity evolution of Subject 3 is shown in Fig 4 (Subject 1 and Subject 2, S1 Fig). The degree of connectivity was highest at the movement execution. Also, the connectivity remained low around 2000 ms before the movement onset. Connectivity gradually increased even before the movement onset, even though it decreased according to the movement offset. All three subjects showed the consistent temporal dynamics of 1M and DLPFC-1M networks, except for the DLPFC network. DLPFC network dynamics of Subject 2 showed similar temporal dynamics of 1M and DLPFC-1M. However, Subjects 1 and 3 showed that the temporal dynamics of DLPFC network remained low regardless of movement states.

**Fig 5:**
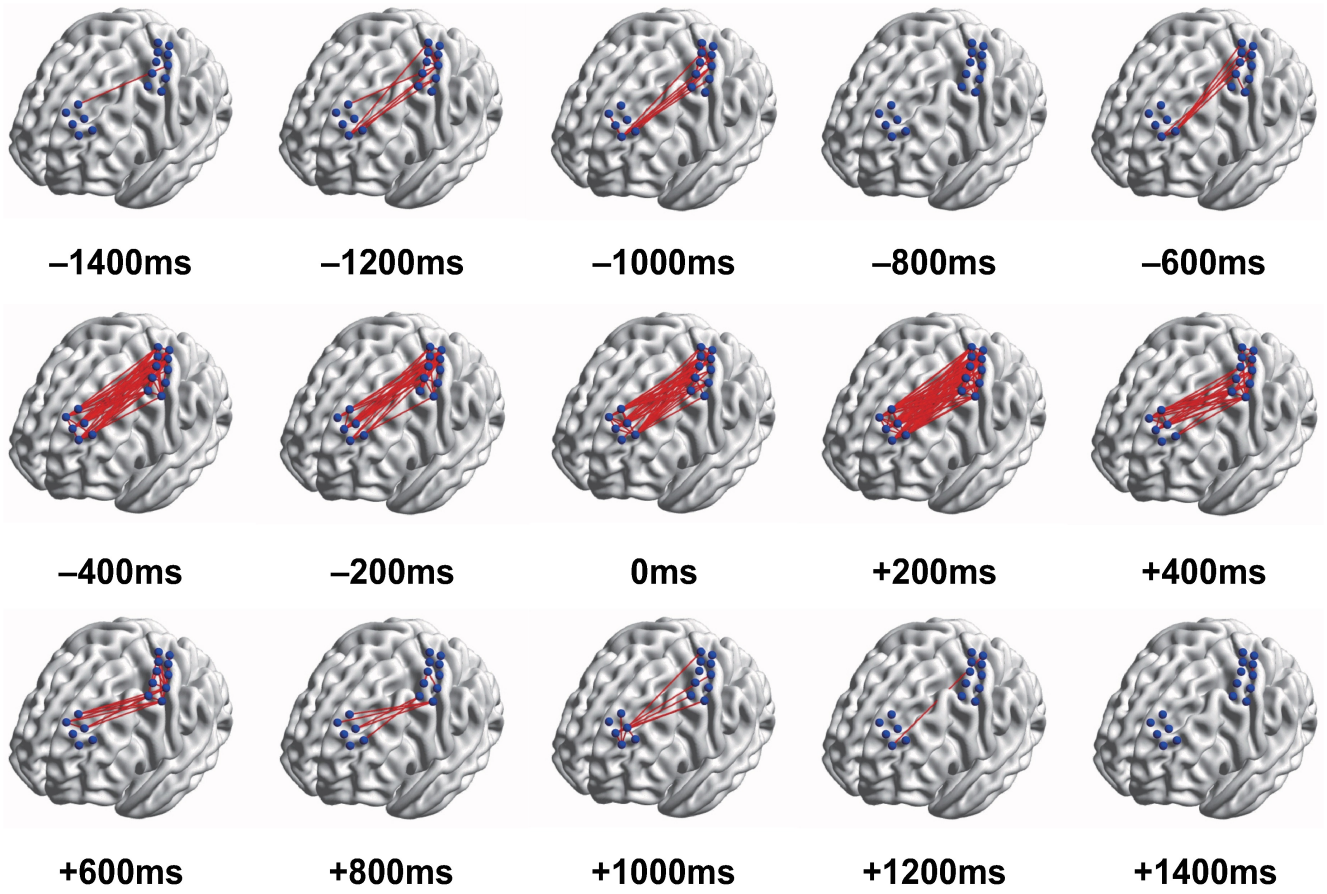
Intra- and inter-regional connectivity evolution in time (Subject 3). The strongly connected pairs are presented as red lines on a cortical surface model at every 200ms. It clearly shows different temporal connectivity dynamics over the movement time.

We also demonstrated the temporal change of local connectivity patterns in every pair with an interval of 200 ms (Fig 5). BrainNet Viewer was used to visualizing the temporal change of intra- and inter-regional connectivity in DLPFC, 1M, and DLPFC-1M networks [29]. Strong connectivity over 0.8 of normalized MI between two electrodes was evident (Fig 5, line).

The strength of connectivity distinctly showed different trends over the movement time (Figs 4 and 5). In the IS, there was little connectivity between DLPFC and 1M. The strength and connections of connectivity increased gradually from -600 ms to 600 ms. Since then, it decreased gradually.

### Prediction of movement intention with the 1M network

Conventionally, 1M has been used to be the main area to predict movement. We tested the classification accuracy in the 1M network. All connection pairs within 1M area were used for constructing a feature to predict movement intention. The mean overall classification accuracy obtained from 10 times cross-validation procedure for each subject. The mean classification accuracy averaged over subjects was 86.2% (Table 2).

**Table 2:** Classification accuracy.

	1M	DLPFC and 1M
Subject 1	91.3 ± 0.5%	94.9 ± 0.4%
Subject 2	78.1 ± 0.3%	93.0 ± 0.2%
Subject 3	89.2 ± 0.4%	94.2 ± 0.3%
Total average	86.2%	94.0%

### Prediction of movement intention with the 1M, DLPFC, and DLPFC-1M networks

**Fig 6:**
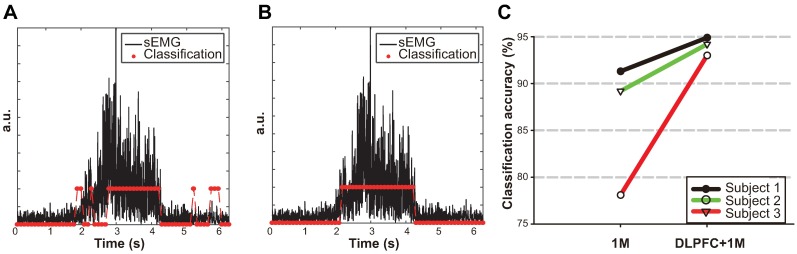
Prediction of movement intention. Two classifiers were evoked to predict movement intention (i.e., 1M network only and DLPFC-1M network). (A) The prediction result of movement intention with only 1M network. (B) The prediction result of movement intention with both DLPFC and 1M network. (C) The classification accuracy of three subjects in two conditions, 1M and DLPFC-1M network.

We also investigated the contribution of DLPFC with regard to predicting movement intention. To do this, we compared two different classifiers for the 1M only network and the networks of DLPFC and 1M. In the case of 1M, DLPFC, and DLPFC-1M network classifications, all connection pairs within and between DLPFC and 1M areas were used to construct a feature to predict movement intention. The mean overall classification accuracy was obtained from 10 cross-validation procedures for each subject. When we used both DLPFC and 1M areas, the mean classification accuracy across all subjects improved around 7.8% compared to the 1M network only (Fig 6C). The three subjects had 94.9%, 93.0%, and 94.2% classification accuracy, respectively (Table 2). Fig 6 depicts the classification result of Subject 2 according to the temporal change of the movement.

## Discussion

This study clearly shows that movement intention can be successfully predicted by connectivity dynamics of DLPFC and 1M networks. The implication of these results is that in addition to being discriminable with spectral power, that functional connectivity also allows the detection of user intention. Through this, we can selectively operate a BMI system and prevent unintended movement prediction. Some previous studies successfully estimated three-dimensional arm movement trajectory and types by the characteristic features of neural signals, using models typically built and validated based on data from well-designed trial periods during which a subject actively performed specific movement tasks [1–3, 30]. However, one study reported that severe prediction error occurred during idle periods due to parameters optimized for tasks [4]. This prediction error could be a critical issue to hamper real-world prosthetic devices for patients because it would lead to unexpected movements, sometimes resulting to accidents. Furthermore, most BMI users have difficulty performing independent activities. Someone else turned the BMI system on and off. This is a burden to BMI users. To solve these problems, we took a new approach to predict movement intention using brain connectivity.

Brain connectivity has become recently considered as a new promising approach to investigate the brain [31]. Neural communication can be reflected in brain connectivity. Since connectivity approach includes interactions or statistical dependencies of two different sites, it may have different information and more features compared to neuronal firing or power analysis. It is advantageous to investigate various functional brain circuits. Furthermore, an ECoG approach is much more advantageous to estimate brain connectivity than a microelectrode approach since it covers larger brain area than microelectrodes.

A previous microelectrode study on the primary motor cortex found that the activity pattern well-distinguished rest from movement task in two monkeys (*Macacamulatta*) [4]. Microelectrode recordings provide the electrophysiological response of single neuron. Although the single-unit activity has the finest spatial resolution in a microscopic level, it would be limited when investigating neuronal communication in a macroscopic neocortical network. On the other hand, ECoG would be advantageous for the analysis of the neural communication between distinct brain regions interconnected by inter-regional pathways. Moreover, the microelectrode approach may have a risk of infection due to its invasiveness and a potential limitation of long-term durability because of an immune response to microelectrodes [32]. The ECoG-based system had much more durable and stable than single unit activity based system. We achieved high success rates of prediction using brain connectivity analysis. Thus brain connectivity might be an excellent approach to predict movement intention. It is also vital to elucidate information flow within and between networks.

Analysis of ECoG revealed that the connectivity in the gamma band increased most prominently in AS compared to other frequency bands. The MI of the gamma band then was used for constructing a feature to predict brain states. Many studies have been conducted to investigate gamma rhythms. Gamma rhythms could be generally decomposed into low-gamma (30–70 Hz) and high-gamma (>70 Hz) although the precise frequency ranges vary across studies. Although low-gamma is well known for being modulated by sensory input and internal processes such as sensory processing and memory, high-gamma activity shows different characteristics compared to the low-gamma activity [33–37]. There are some studies described high-gamma power change is related to motor movements [38–42]. These authors suggested that high-gamma oscillatory activities at the cortical level would be mechanistically involved in determining motor behavior and could even improve motor performance. In this study, we approached the prediction of movement intention with network dynamics, not spectral power changes. The result of this study indicates that the lower gamma activities generated from both DLPFC and 1M potentially communicate with each other and that the lower gamma activity in DLPFC may contribute to the motor behavior. The intra- and inter-regional connectivity of lower gamma band vary depending on the movement time (Fig 5). The lower gamma oscillation generated from DLPFC and 1M may play a crucial role in motor control through an information transmission.

The temporal change of the connectivity in the DLPFC-1M network before the movement onset is very similar to the readiness potential (RP) (Fig 4). RP refers to a brain activity leading up to voluntary muscle movement, which is generated in the motor and prefrontal cortices. RP is well known for a manifestation of cortical contribution to the pre-motor planning of volitional movement [43]. RP but also beta and mu event-related desynchronization (ERD) features lead a movement onset, and end after the movement offset over a very broad region. In the previous studies, these features and spectral analysis were used to predict motor intention [8, 11]. In this study, we focused on temporal network dynamics to predict motor intention. Information exchange gradually increased prior to the movement onset, peaked during movement, and decreased after movement offset for both intra-connectivity of 1M and inter-connectivity of DLPFC-1M. The findings imply that the connectivity between DLPFC and 1M may also reflect the planning of movement, such as RP[44].

The classification performance was improved when we used the connectivity of both DLPFC and 1M networks as a feature compared to using the connectivity in the 1M network only (Table 2). This implies that DLPFC is crucial in predicting movement intention, which is at least comparable to 1M. DLPFC has preferential connections to the motor system, such as the supplementary motor area, the premotor area, and the 1M. DLPFC plays a key role to exert control over behavior, and it has a central integrative function for motor control and behavior [22, 23, 25]. Despite these findings, most BMI studies focused only on the 1M. However, we suggest that 1M and movement-related areas are crucial in the movement, which must be guided by internal states or intention. In this study, we show clearly that DLPFC actively communicates with 1M. The connection strengthens during movement planning and execution. This implies that DLPFC is largely engaged in the movement planning and execution.

## Conclusions

This study clearly shows the movement intention can be successfully predicted by connectivity dynamics which may reflect changes in movement states. It refers that the users of BMI can control the system by their free will. We firmly believe that this is a very important finding to prevent unexpected error and to develop practical BMI systems. However, there still remains some concerns about implementing a real-time BCI such as timing and subject adaptation. Moreover, network dynamics needs to be directly compared to conventional models based on spectral power analysis to determine whether functional connectivity is better, worse, or no different.

For further studies, our approach can be applied to on-and-off control for a BMI system predicting the movement trajectory. With our approach, unintended robot arm movement may be prevented during the IS and BMI users can selectively and independently control the BMI system. We also expect that the proposed approach can be applied to a multi-mode BMI system: a unified BMI system with various control functions, such as robot arm, wheelchair, and keyboard. In the real world, BMI users may need one or more functions for their active life. We successfully classified only movement intention state. However, it might be expanded to other functions if we estimate the connectivity in language, attention, and memory networks.

## Supporting information

S1 Fig Temporal connectivity dynamics in DLPFC, 1M, and DLPFC-1M networks of Subject 1 and Subject 2. Temporal connectivity dynamics represents respectively according to movement onset and offset. Pink vertical dotted line denotes movement onset (left panel) and movement offset (right panel). (A, B) Subject 1. (C, D) Subject 2.
(TIF)

S1 Data The ‘S1 Data’ consists of ECoG data and sEMG data. ECoG data was recorded from selected electrodes on 1M and DLPFC. sEMG data was recorded on the opponens pollicis.
(MAT)
