# Supplementary figures and images for: Prediction of movement intention using connectivity within motor-related network: An electrocorticography study
Source: PLoS One. 2018 Jan 24;13(1):e0191480. doi: 10.1371/journal.pone.0191480 (PMC5783365; doi:10.1371/journal.pone.0191480)

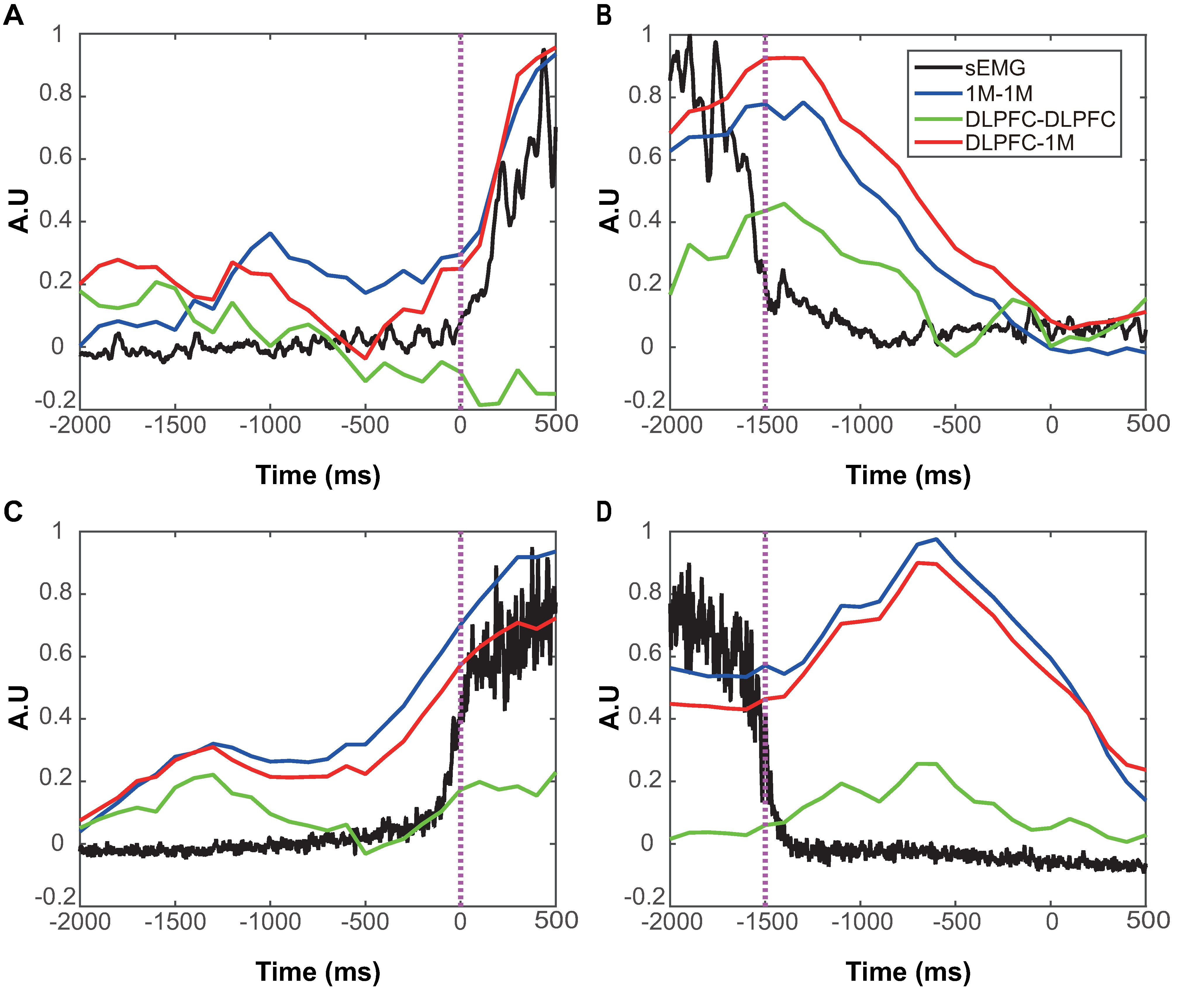

Supplement: S1 Fig — Temporal connectivity dynamics represents respectively according to movement onset and offset. Pink vertical dotted line denotes movement onset (left panel) and movement offset (right panel). (A, B) Subject 1. (C, D) Subject 2. (TIF) [file pone.0191480.s001.tif]
